# Increases In Women's Political Representation Associated With Reductions In Child Mortality In Brazil

**DOI:** 10.1377/hlthaff.2019.01125

**Published:** 2020-07-01

**Authors:** Philipp Hessel, María José González Jaramillo, Davide Rasella, Ana Clara Duran, Olga L. Sarmiento

**Affiliations:** Alberto Lieras Camargo School of Government, University of the Andes, in Bogotá, Colombia; Inter-American Development Bank in Washington, D.C; Institute of Public Health, Federal University of Bahia, in Salvador de Bahia, Brazil; University of Campinas, in Campinas, Brazil; and a research fellow at the Center for Epidemiological Studies in Nutritionand Health, University of São Paulo, in São Paulo, Brazil; School of Medicine, University of the Andes

## Abstract

We assessed the effects of female political representation on mortality among children younger than age five in Brazil and the extent to which this effect operates through coverage with conditional cash transfers and primary care services. We combined data on under-five mortality rates with data on women elected as mayors or representatives in state and federal legislatures for 3,167 municipalities during 2000–15. Results from fixed-effects regression models suggest that the election of a female mayor and increases in the shares of women elected to state legislatures and to the federal Chamber of Deputies to 20 percent or more were significantly associated with declines in under-five mortality. Increasing the political representation of women was likely associated with beneficial effects on child mortality through pathways that expanded access to primary health care and conditional cash transfer programs.

One key aim of the Sustainable Development Goals is to ensure women’s full and effective participation in decision making in political, economic, and other forms of public life. This includes providing women with equal opportunities for leadership. Despite progress on some indicators, most countries are far from reaching gender parity in politics, with women accounting for only 25 percent of nation-al parliament members worldwide.^1^

Increasing women’s political representation should be regarded as a goal in its own right. However, a growing body of evidence suggests that doing so may positively affect policy outcomes, especially in relation to access to and quality of child care and health care, as women bring a different set of attitudes, priorities, and styles of leadership to the table.^2–4^ For those reasons, women’s political empowerment has gained attention as a potentially important factor for improving population health.^5^ Support for this view is based on evidence that female politicians tend to place a higher priority than men do on the provision of public goods, including health and education.^2^ Greater involvement of women in political decision making may also be positively related to larger investments in policies related to education, health care, and social welfare services.^6^ When women are represented in government, they very often occupy positions in the ministries of health and social affairs,^7^ which can lead to greater investment in those domains. This investment helps counteract unequal social structures and unequal access to social, cultural, and economic resources, which can lead to a social patterning of disease by shaping exposures to hazardous living and working conditions, discrimination, and violence.^8^

Empirically, gender equity as well as women’s political representation are positively associated with many health outcomes, including life expectancy.^9^ Specifically, increasing women’s political and economic empowerment may positively affect children’s health through a series of pathways. For example, increasing gender equity is generally associated with the greater participation of women in education and the labor market.^11^ This may increase mothers’ knowledge of economic resources and thus facilitate their access to maternal health care services and good child nutrition.^12^ Studies have shown that women’s empowerment is positively associated with the provision of child care services,^4^ prenatal care visits, skilled attendance at birth, vaccination, and improved nutritional status.^13^

Despite compelling reasons why increases in women’s political representation should improve child health, as well as a robust association between these two dimensions of representation and health, two important knowledge gaps remain. First, the few existing studies on the topic that have used longitudinal data to address issues of confounding have been based on pooled samples from many countries,^9,10,12^ which makes it difficult to determine what specific mechanisms explain the relation between women’s political representation and health. Second, because of the reliance on country-level data of most related studies, it remains largely unknown at what level of political decision making female representation matters for child health.

Using longitudinal data from 2000–2015 for municipalities in Brazil, this study aimed to assess the associations of female political representation with under-five mortality rates and to investigate pathways that might link female participation in politics to the rollout of both the Bolsa Famí lia program (BFP), a conditional cash transfer program, and the country’s primary health care program, the Estratégia de Saúde da Família (ESF). Brazil provides an appropriate case for studying these issues for several reasons. First, the country has experienced a large decrease in child mortality in the past twenty years, accompanied by substantial investments in social and primary health care programs during the presidency of Luiz Inácio Lula da Silva, from the Workers’ Party. Studies have shown that both programs have led to important improvements in child health.^14^ Given evidence that female politicians are generally more likely than male politicians to support such programs,^16^ this represents a plausible pathway through which female political representation may affect child health in Brazil. Second, the availability of detailed longitudinal data on child mortality and BFP and ESF coverage, as well as election outcomes, makes it possible to empirically evaluate on what level female political representation and decision making matter for child mortality, and to what extent investments in social programs may explain this relationship.

## Study Data And Methods

### Data

To analyze associations of women’s political empowerment and child mortality, we constructed a longitudinal data set with yearly data for 2000–15 based on several publicly available databases, using the municipality as the unit of analysis. From a total of 5,565 municipalities in Brazil, we selected a subset of 3,167 municipalities that have been identified as having a death registry system with fewer than 10 percent of deaths missing.^17^

### Under-Five Mortality Rate

Mortality among children younger than age five was the dependent variable. Information on the number of deaths of children in this age group as well as the number of live births in each municipality and year in the study period was obtained from the Ministry of Health.^18^ We then calculated the under-five mortality rate per 1,000 live births for each municipality and year.

### Women in Political Office

We constructed a set of indicators to measure women’s representation in politics using data from the Superior Electoral Court.^19^ Female political representation was captured by three variables.

The first variable was whether a municipality had elected a female mayor. Mayors in Brazil play an important part in the delivery of public services, especially with regard to health care, social welfare programs, and education. Although the lion’s share of funding for such programs is provided through federal transfers, municipalities decide on exact resource allocations as part of a wider decentralization strategy.^20^ In particular, decentralization has been credited with a substantial improvement in access to public health care, mainly in the form of the ESF.^21^ Municipalities also carry out several aspects of the BFP, especially by managing the enrollment process, monitoring whether recipients comply with the program’s health- and education-related requirements, and selecting people to receive other complementary social services.^22^

The second variable was the percentage of elected female representatives in the state legislature. States play mainly a coordinating and planning role for their municipalities in the provision of health care. However, in less developed regions, state governments often play a more important role, as municipalities may lack the technical expertise and administrative capacity to effectively implement the ESF.^23^ States also provide technical support and training for municipalities for the delivery of the BFP.^22^ It has been argued elsewhere that the federal government—which has been controlled by the Workers’ Party since 2003—has purposely tried to sideline state legislatures and executives regarding the delivery of key social programs in an effort to receive direct credit for the programs.^24^ In terms of decision-making processes, mayors in Brazil have the functions of city managers, performing not only political but also administrative duties and being the key decision makers for municipal development.^25^ While municipal legislatures are in charge of local legislative matters, mayors are responsible for most local administrative matters, including resource allocation.

The third variable was the percentage of elected female representatives in the federal Chamber of Deputies, which has 513 members. The Chamber of Deputies is the lower house of Brazil’s Congress, and it must approve each new law. Since the federal government is the primary funder of all major social programs as well as education and health care in Brazil, the Chamber of Deputies plays a uniquely important role—not only in setting funding levels but also in the design of each program. Because of the significant underrepresentation of women in the Senate, of which only eleven of eighty-one members were women as of 2014 (during our study period), we omitted the Senate from our analyses.

Municipal elections take place every four years (there were elections in 2000, 2004, 2008, and 2012 during our study period). General elections (in which the governors of the twenty-seven federal states and members of the state assemblies, as well as the president and members of Congress) also take place every four years but on a different schedule (2002, 2006, 2010, and 2014 in our study period).

Female political representation in the state legislature and the federal Chamber of Deputies was included in our analyses in the form of a categorical variable that captured female representation at the following percentages: 0–9 percent, 10–19 percent, and 20 percent or more of the elected representatives in each respective session. This is in line with previous studies showing that a critical mass of around 20 percent of elected female politicians may be required in a legislative body for significant change to occur.^26^

While the presence of a female mayor is municipality specific, the percentage of women represented in the federal Chamber of Deputies and state legislature differs only according to the state in which a municipality is located.

We assigned the results of a given election to the year in which the election took place as well as to each of the following years until the next election for that office took place.

### Covariates

We included a comprehensive set of covariates to control for the socioeconomic characteristics of the population in each municipality. The covariates we used included information on individuals’ income (percentage of peo-ple who earned at least the minimum wage) and education (percentage of people with less than a high school education), percentage of households with access to regular garbage collection and running water, and women’s education (the illiteracy rate) for single years between 2000 and 2010. They were calculated using an interpolation method that combined census data with information from regional and national household surveys, according to published methods.^27^ Values for the years 2011–15 were linearly extrapolated.

**Female political representation matters for child health not only at the national or state level, but also at the local level**

We included information on yearly municipal-level BFP and ESF coverage (defined as the percentages of eligible individuals or households registered in each program). As part of the BFP, impoverished families (who make up approximately 25 percent of the population) receive a monthly cash benefit that varies based on their conditions of vulnerability (the national average was US$50 as of July 2018) if families meet certain criteria related to children’s school attendance and regular health checkups. Studies have shown that conditional cash transfer programs such as this have contributed to substantial improvements in child health and mortality in Brazil.^15^ The ESF is a public health program aimed at improving access to primary care across the country. Previous studies have found that the ESF was associated with reductions in child mortality,^28^ overall mortality, and unnecessary hospitalizations.

### Statistical Analyses

To assess the effects of changes in female political representation on under-five mortality and social protection programs in each municipality, we used linear fixed-effect regression models for panel data. Similar types of analyses, in combination with municipality-level data, have been used in various previous studies to assess the effects of the BFP, primary health care coverage (in the form of the ESF), and decentralization on health and mortality in Brazil.^14^ The advantage of using these models is that they effectively control for all time-invariant differences across municipalities and use only within-municipality variation over time to identify the effects of changes in women’s political representation on child mortality. Although differences in levels of child mortality existed between municipalities that ever elected female mayors and those that never did so during the years we studied, we found no significant differences in pretreatment time trends between both groups of municipalities when we controlled for covariates.

To establish whether female political representation affected child mortality and then to consider how BFP and ESF coverage could represent a plausible pathway linking coverage with both programs and child mortality, we proceeded as follows: First, we regressed under-five mortality on indicators of female political representation to assess whether increases in the latter were associated with changes in the former. Second, we regressed municipality-level BFP and ESF coverage on under-five mortality to assess whether the former was significantly associated with the latter. Third, we regressed BFP and ESF coverage on indicators of female political representation to assess whether increases in the latter were associated with higher program coverage. Lastly, we assessed whether the inclusion of BFP and ESF coverage as covariates mediated the effect of female political representation on child mortality.

All analyses used municipality-level cluster-robust standard errors to adjust for potential heteroskedasticity and autocorrelation. More information about the regression models is in online appendix exhibit Al.^30^

### Limitations

Many previous studies that focused on the effects of female participation in politics on health outcomes have relied on country- or state-level data. The strength of our study was its use of smaller geographic units (that is, municipalities), which limited potential ecological fallacy or exposure misclassification. However, the study had several limitations.

First, we could not rule out the presence of ecological fallacy. However, our finding of a significant effect of greater representation of women on child mortality at the municipality level strengthened our confidence in our other findings. Readers should note that 75 percent of the municipalities in our sample had fewer than 25,000 inhabitants.

Second, while our results were robust to a series of alternative specifications and controlled for a comprehensive set of covariates at the municipality level, other unobserved factors could have affected trends in child mortality. Our empirical strategy included fixed effects for municipalities and calendar years as an additional robustness check, thus effectively controlling for all time-invariant variation across space and consistent secular trends over time.

## Study Results

### Descriptive Findings

Mortality among children younger than age five per 1,000 live births declined from 25.1 in 2000 to 13.6 in 2015 in the municipalities in the sample (exhibit 1). The percentage of female mayors increased from around 4.5 percent to 9.7 percent. The shares of women in state legislatures or the federal Chamber of Deputies overall did not increase over time, remaining around 10 percent and 7 percent, respectively. However, trends varied substantially across municipalities. For example, in the decile of municipalities with the largest increase in the share of women in the state legislature from 2000 to 2015, the percentage increased from 8 percent to 26 percent (data not shown). Average municipality-level BFP coverage increased from 9.6 percent in 2000 to 15.3 percent in 2015, while ESF coverage increased from 25.2 percent in 2000 to 54.7 percent in 2015 (exhibit 2).

### Associations of Female Political Representation and Under-Five Mortality

When we controlled for municipalities’ sociodemographic characteristics, having elected a female mayor was associated with a significant decrease in the under-five mortality rate in a municipality by 0.027 percentage points (exhibit 3) (95% confidence interval (Cl): –0.050, –0.004; data not shown). Furthermore, having a share of women in the state legislature that was 20 percent or higher was associated with a decrease in under–five mortality of 0.038 percentage points (95% Cl: –0.075, –0.001). Also, increases in the share of women elected to the federal Chamber of Deputies from 0–9 percent to 10–19 percent or to 20 percent or higher were associated with significant decreases in child mortality of 0.038 percentage points (95% Cl: –0.057, –0.001) and 0.072 percentage points (95% Cl: –0.122, –0.022), respectively.

### Association of Social Protection Program Coverage With Under-Five Mortality

Appendix exhibit A2 shows that increases in municipality-level BFP and ESF coverage were significantly associated with reductions in under-five mortality.^30^ On average, an increase of 1.0 percent in municipality-level BFP coverage was associated with a decrease of 1.0 percent in under-five mortality (coefficient: –0.01; 95% Cl: –0.001, –0.017), and an increase of 1.0 percent in ESF coverage was associated with a decrease of 0.8 percent in child mortality (coefficient:–0.008; 95% Cl: –0.005, –0.011).

### Association of Female Political Repre-Sentation with social Protection Program Coverage

Increases in the percentages of women elected as municipal or federal representatives, as well as the election of female mayors,were significantly associated with increases in BFP coverage (exhibit 4). Furthermore, electing a female mayor was significantly associated with a 1.9 percent increase in ESF coverage within a municipality. In addition, increases in the percentage of women elected to the state legislature(from 0–9 percent to 20 percent or higher) were associated with a 4.2 percent increase in ESF coverage, and increases in the percentage of women elected to the Chamber of Deputies (from 0–9 percent to 10–19 percent) were associated with a 1.2 percent increase in ESF coverage.

When we controlled for BFP and ESF coverage, the association between the indicators of female political representation and under-five mortality became slightly attenuated: Once representation increased to 20 percent or higher of women in state legislatures, the association was no longer significant (exhibit 3). At the same time, the association between increases in BFP and ESF coverage with decreases in under-five mortality became weaker, in comparison to the models that did not control for BFP and ESF coverage (exhibit 3).

### Robustness Analyses

We estimated additional models to assess the robustness of our results to alternative specifications. First, we estimated models that included all 4,943 municipalities for which data were available. Second, we included fixed effects for calendar year, as well as municipality-year interactions. Third, we included controls for all parties’ vote shares in each municipality election. Last, we used the infant mortality rate (ages 0–1 years) as a dependent variable. As the results in appendix exhibit A3 show,^30^ these estimates did not change the qualitative interpretation of our findings.

## Discussion

Our results suggest that both electing female mayors and increasing the share of female members of state legislatures and the federal Chamber of Deputies were associated with significant reductions in child mortality. We also found that increases in female elected officials were associated with significant increases in municipal BFP and ESF coverage, and coverage expansions of both programs were in turn significantly associated with reductions in child mortality. In sum, increases in BFP and ESF coverage resulting from of increases in female political representation at different levels of government and greater participation in decision making represent a plausible pathway from female political representation to reduced child mortality.

Although several previous studies have found positive correlations between female political representation and child health using cross-sectional data,^9^ few studies have assessed this relationship using longitudinal data. While our results are generally in line with those studies,^6,10,31–33^ one of our key findings is that female political representation matters for child health not only at the national or state level, as previously shown in the case of the United States,^31^ but also at the local level. The importance of local politicians in Brazil and many other Latin American countries has been steadily increasing as a result of wide-ranging decentralization trends—which have resulted in the transfer of many public services, including health care, to subnational governments.^34^

The relationship between female political empowerment and child health likely involves multiple pathways beyond those we evaluated. However, plausible direct pathways include improvements in access to and quality of education among women, female labor-force participation, and access to economic resources.^35^ Increased political empowerment of women may also have positive effects on access to contraceptive methods, and it may lead to increases in age at first marriage and reduce teenage pregnancy, thus leading to better birth outcomes and declines in child mortality. Studies have also shown that increases in gender equity and political empowerment are associated with more prenatal care visits and vaccinations.^12^ In Brazil one study using a quasi-experimental design found that municipalities that elected female mayors received more federal discretionary transfers, had lower levels of corruption, hired more public employees, and had higher rates of prenatal care.^36^ Those findings are in line with the fact that elected female politicians in Brazil are generally more likely than their male counterparts to focus on social issues—including health, education, and family—in their legislative behavior.^16^ Furthermore, evidence also suggests that women elected as mayors in Brazil are more likely to appoint another woman to lead the local health agency, and in turn those other women are empirically more likely to support women-friendly policies such as those related to health care services and birth and day care centers.^37^

While previous studies have shown positive effects of conditional cash transfer programs on child health in Brazil,^15^ our results suggest that increases in female political representation were associated with higher BFP coverage rates. Expanding the program’s coverage likely has direct effects on child health as a result of the mandatory health checkups, vaccinations, and growth monitoring required for children in BFP recipients’ families. With its monthly cash transfers, the program may also have other positive effects on child health through improved quantity and quality of nutrition. Since the program’s launch in 2003, there has been pressure from women’s movements to include a gender perspective in the BFP design, which contributed to its women-focused orientation. This includes handing out the cash benefit directly to mothers, which increases women’s decision-making power in the household.^38^ Women elected to municipal assemblies have the potential to act effectively on policies that can improve the lives of families and communities and can positively influence policies aimed at meeting the needs of women and children.^39^ To receive federal funds to implement policies related to social welfare, primary health care, and elementary education, municipalities need to sign a covenant directly with the federal government. The covenant requirs municipalities to adhere to certain conditions; follow federal guidance; and meet efficiency or target indicators, or both.^24^ The provision of primary health care and elementary education in Brazil falls primarily under the jurisdiction of municipalities. For the BFP, municipalities are also responsible for identifying eligible families and enrolling them in the program, as well as checking their adherence to the program’s health and education requirements.^39^

Besides the positive effect of female political representation on the rollout of the BFP, our results also suggest that electing female mayors has a positive effect on ESF coverage. Several studies have found a positive effect of municipality-level ESF coverage on various health indicators.^14^ It is very plausible that increasing women’s involvement in political decision making had a positive effect on the ESF’s implementation within municipalities, because of the important role played by women’s groups in the reform of the health sector since the end of Brazil’s authoritarian military dictatorship in 1985.^20^ While funds for the ESF come from the federal government, the public health system is highly decentralized, with municipalities being responsible for the allocation of resources as well as the provision of services. Furthermore, the federal government provides financial incentives to municipalities for implementing the ESF. Women may directly benefit from increased ESF coverage, especially from its services related to prenatal care and family planning activities. It is likely that these benefits incentivize female politicians to push for comprehensive implementation of the program and work to prevent corruption within the system.^36^

## Policy Implications

Despite significant progress and expansions in public health and social security coverage, Brazil has one of the world’s lowest levels of female representation in politics. Data from the Inter-parliamentary Union show that Brazil is 152 out of 193 assessed countries in terms of female participation in both the lower and upper houses of the legislature.^1^ In 2020 only 14 percent and 13 percent of the lower and upper houses, respectively, were women. In contrast, Mexico, an upper-middle-income Latin American country, is 5 out of 193, and 48 percent and 49 percent of that country’s lower and upper houses, respectively, were women in 2020.^1^ To fight such gender disparities, in 2009 a law appended to a previous Brazilian law stated that every political party should have a minimum of 30 percent and a maximum of 70 percent of candidates of each gender.^41^ Although the share of female candidates has increased in the past years in Brazil, the percentage of elected women has not shown a corresponding increase. A possible explanation lies in the unequal distribution of funding by parties between female and male candidates.^42^ Another is discrimination against women by voters.^43^ As the results of this study further highlight, increasing the de facto representation of women in politics should be a key policy priority in Brazil.

## Conclusion

The results of this study suggest that the lack of equitable female political representation could impede progress toward achieving not only the Sustainable Development Goal of gender equity but also the other goals of reducing child mortality and poverty. Progress toward equal representation of women in political decision making in Brazil may be accompanied by sizable improvements in child mortality and life expectancy, partly as a result of increased public funding and access to primary health care services as well as conditional cash transfer programs. Improving female participation at the federal and local levels may also lead to greater support for other pressing policies that have been associated with better child health outcomes, such as access to child care, support for family leave that can be split between parents, and creating more supportive environments for breast-feeding. Although gaining a better understanding of the intermediate mechanisms that link women’s political representation to health is important, future research should also aim to increase understanding of the political processes that produce such outcomes. The fields of public health and social epidemiology increasingly acknowledge the roles played by political and economic contexts in social determinants of health. The results of this study highlight the importance of using a broader definition of determinants of health that delineates the political and economic pathways for improving population health around the globe.

## Figures and Tables

**Exhibit 1:**
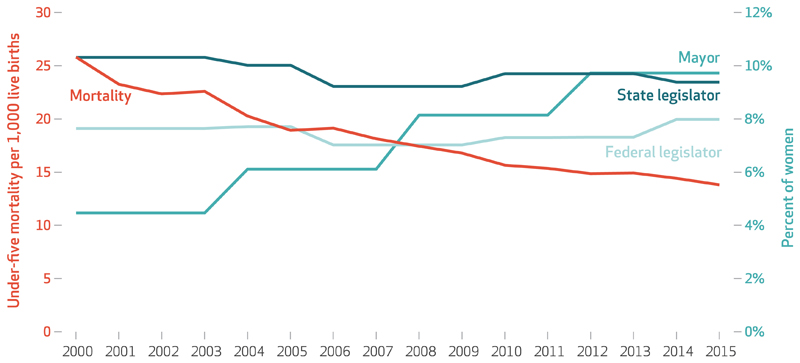
Under-five mortality per 1,000 live births and percent of women in selected political positions in Brazil, 2000–15 **SOURCE** Authors' analysis of data for 3,1 67 municipalities from the Brazilian Ministry of Health and data from the Superior Electoral Court, **NOTES** The municipalities had death registries with fewer than 1 0 percent of deaths missing, as explained in the text. Federal legislators are those elected to the Federal Chamber of Deputies, Brazil's lower legislative house.

**Exhibit 2:** Descriptive statistics for brazil in 2000 and 2015

	Average across municipalities	
	As of 2000	As of 2015
**OUTCOME**		
Under-five mortality rate (per 1,000 live births)	26.10	13.65
**INDEPENDENT VARIABLES: FEMALE POLITICAL REPRESENTATION**		
Female Mayors Overall	4.91 %	11.47%
Women holding political office		
State Legislators (Elected In Most Recent State Election)	12.52%	9.92%
0–9%	51.06	52.31
10–19%	42.18	47.69
20% or more	6.75	7.21
Members of the federal chamber of deputies	7.49	7.68
0–9%	68.68	85.55
10–19%	27.71	9.77
20% or more	3.61	4.68
COVARIATES		
Bolsa Familla Program (BFP) Coverage	9.64%	15.30%
Estratégia de Saúde da Família (esf) coverage	25.24	54.71
People Earning At Least The Minimum Wage	19.59	47.30
Households With Regular Garbage Collection	82.79	81.34
Households With Running Water	66.95	70.57
People With Less Than A High School Education	17.27	12.66
Women Who Were Illiterate	10.11	5.35

**SOURCE** Authors' analysis of data for 3,167 municipalities from the brazilian ministry of health; the superior electoral court; and guanais FC. municipal-level covariates of health status in brazil [note 27 in text), **NOTES** The information refers to the average of all municipalities in the sample as of 2000 and 201 5, respectively. the municipalities had death registries with fewer than 1 0 percent of deaths missing, as explained in the text.

**Exhibit 3:** Estimated relationships between women holding political office and under-five mortality in Brazilian municipalities, 2000–15

	Under-five mortality per 1,000 live births (log)	
	Controlling For Municipality Socioeconomic Characteristics	Controlling additionally for BFP and ESF coverage
**INDEPENDENT VARIABLES: FEMALE POLITICAL REPRESENTATION**		
Female Mayor In The Municipality	–0.027**	–0.021**
Women Holding Political Office		
State Legislators (ref: 0–9%)		
10–19%	0.007	0.004
20% or more	–0.038*	–0.023*
Members Of The Federal Chamber Of Deputies (ref: 0-9%)		
10–19%	–0.038****	–0.018**
20% or more	–0.072***	–0.079****
**COVARIATES**		
Percent Of People Earning At Least The Minimum Wage	–0.002**	–0.002***
Percent Of Households With Regular Garbage Collection	–0.001****	–0.001****
Percent Of Households With Running Water	–0.002****	–0.002****
Percent Of People With Less Than A High School Education	0.007****	0.007****
Percent Of Women Who Were Illiterate	0.019****	0.019****
year	–0.020****	–0.026****
Coverage By Any Social Protection Program		
BFP	—^a^	–0.001****
ESF	—^a^	–0.002****

**SOURCE** Authors ' Analysis Of Data For 3,167 Municipalities From The Brazilian Ministry Of Health; The Superior Electoral Court; And Guanais Fc. Municipal-Level Covariates Of Health Status In Brazil (Note 27 In Text), **Notes** The Exhibit Shows The Results From A Linear Regression Model For Panel Data Including Fixed Effects For Municipalities. The Dependent Variable [Mortality For Children Younger Than Age 5 Per 1,000 Live Births) Was Log-Transformed. A One-Unit Change In A Covariate Thus Indicates The Associated Change In Terms Of Under-Five Mortality. There Were 49,722 Observations Of Municipality-Years. The Municipalities Had Death Registries With Fewer Than 1 0 Percent Of Deaths Missing, As Explained In The Text. For All Elections, Usually Taking Place Every Four Years, We Assigned The Results Of A Given Election To The Year In Which The Election Took Place As Well As Each Of The Following Years Until The Next Respective Election; See The Text Or More Details. BFP Is Bolsa Famllia Program. Esf Is Estrategia De Saude Da Familia. aNot Applicable. *p < 0.10**p < 0.05 ***p<0.01****p<0.001

**Exhibit 4:** Estimated relationships between women holding political office and social protection program coverage in Brazilian municipalities, 2000–15

Independent variables: female political representation	BFP coverage(%)	ESF coverage(%)
Female mayor in the municipality	2.449****	1.907****
Women holding political office		
State legislators (ref: 0-9%)		
10–19%	0.535****	0.462****
20% or more	–0.366	4.182****
Members of the federal chamber of deputies (ref: 0-9%)		
10–19%	1.173****	1.169****
20% or more	3.773****	–0.567

**source** Authors' analysis of data for 3,1 67 municipalities from the Brazilian Ministry of Health; the Superior Electoral Court; and Guanais FC. Municipal-level covariates of health status in Brazil (note 27 in text), **NOTES** The exhibit shows the results from a linear regression model for panel data including fixed effects for municipalities. The municipalities had death registries with fewer than 10 percent of deaths missing, as explained in the text. The covariates and the number of observations are the same as in exhibit 3. BFP is Bolsa Familia program. ESF is Estrathgia de Saiiide da Familia. **p < 0.05 ****p < 0.001

## References

[1] Inter-Parliamentary Union Women in politics: 2020.

[2] Paxton P, Knnovich S, Hughes MM (2007). Gender in politics. Annul Rev Sociol.

[3] Chaney CK, Saltzstein GH (1998). Democratic control and bureaucratic responsiveness: the police and domestic violence. Am J Pol Sci.

[4] Bratton KA, Ray LP (2002). Descriptive representation, policy outcomes, and municipal day-care coverage in Norway. Am J Pol Sci.

[5] World Bank (2003). Gender equality and the Millennium Development Goals.

[6] Bhalotra S, Clots-Figueras I (2014). Health and the political agency of women. Am Econ J Econ Policy.

[7] United Nations (2017). Women’s political parity slow to grow as UN launches latest “Women in Politics” map [Internet].

[8] Krieger N (2012). Methods for the scientific study of discrimination and health: an ecosocial approach. Am J Public Health.

[9] Shen C, Williamson JB (1997). Child mortality, women’s status, economic dependency, and state strength: a cross-national study of less developed countries. Soc Forces.

[10] Macmillan R, Shofia N, Sigle W (2018). Gender and the politics of death: female representation, political and developmental context, and population health in a cross-national panel. Demography.

[11] Duflo E (2012). Women empowerment and economic development. J Econ Lit.

[12] Pratley P (2016). Associations between quantitative measures of women’s empowerment and access to care and health status for mothers and their children: a systematic review of evidence from the developing world. Soc Sci Med.

[13] Boehmer U, Williamson JB (1996). The impact of women’s status on infant mortality rate: a cross-national analysis. Soc Indie Res.

[14] Macinko J, Marinho de Souza Mde F, Guanais FC, da Silva Simoes CC (2007). Going to scale with community-based primary care: an analysis of the Family Health Program and infant mortality in Brazil, 1999-2004. Soc Sci Med.

[15] Rasella D, Aquino R, Santos CA, Paes-Sousa R, Barreto ML (2013). Effect of a conditional cash transfer programme on childhood mortality: a nationwide analysis of Brazilian municipalities. Lancet.

[16] Miguel LF, Franceschet S, Krook ML, Piscopo JM (2012). Policy priorities and women’s double bind in Brazil. The impact of gender quotas.

[17] Lima EE, Queiroz BL (2014). Evolution of the deaths registry system in Brazil: associations with changes in the mortality profile, under-registration of death counts, and ill-defined causes of death. Cad Saude Publica.

[18] Brazil Ministry of Health Datasus.

[19] Brazil Tribunal Superior Eleitoral Estatisticas.

[20] Collins C, Araujo J, Barbosa J (2000). Decentralising the health sector: issues in Brazil. Health Policy.

[21] Elias PE, Cohn A (2003). Health reform in Brazil: lessons to consider. Am J Public Health.

[22] Linden K, Linder A, Hobbs J, de la Briére B (2007). The nuts and bolts of Brazil’s Bolsa Familia program: implementing conditional cash transfers in a decentralized context [Internet].

[23] Vargas I, Mogollón-Pérez AS, Unger JP, da-Silva MR, De Paepe P, Vázquez ML (2015). Regional-based Integrated Healthcare Network policy in Brazil: from formulation to practice. Health Policy Plan.

[24] Fenwick TB (2009). Avoiding governors: the success of Bolsa Familia. Lat Am Res Rev.

[25] Rocha F, Orellano VIF, Bugarin K (2018). Elected in a close race: mayor’s characteristics and local public finances. Economia.

[26] Swiss L, Fallon KM, Burgos G (2012). Does critical mass matter? Women’s political representation and child health in developing countries. Soc Forces.

[27] Guanais FC (2013). Municipal-level covariates of health status in Brazil: a proposed method for data interpolation. Rev Panam Salud Publica.

[28] Macinko J, Guanais FC, de Fátima M, de Souza M (2006). Evaluation of the impact of the Family Health Program on infant mortality in Brazil, 1990-2002. J Epidemiol Community Health.

[29] Hone T, Rasella D, Barreto ML, Majeed A, Millett C (2017). Association between expansion of primary healthcare and racial inequalities in mortality amenable to primary care in Brazil: a national longitudinal analysis. PLoS Med.

[R30] 30To access the appendix, click on the Details tab of the article online

[31] Homan P (2017). Political gender inequality and infant mortality in the United States, 1990-2012. Soc Sci Med.

[32] Wángnerud L, Sundell A (2012). Do politics matter? Women in Swedish local elected assemblies 1970-2010 and gender equality in outcomes. Eur Polit Sci Rev.

[33] Quamruzzaman A, Lange M (2016). Female political representation and child health: evidence from a multilevel analysis. Soc Sci Med.

[34] Falleti TG (2010). Decentralization and subnational politics in Latin America.

[35] Burroway R (2016). Democracy and child health in developing countries. Int J Comp Sociol.

[36] Brollo F, Troiano U (2016). What happens when a woman wins an election? Evidence from close races in Brazil. J Dev Econ.

[37] Meier KJ, Funk KD (2017). Women and public administration in a comparative perspective: the case of representation in Brazilian local governments. Adm Soc.

[38] De Brauw A, Gilligan DO, Hoddinott J, Roy S (2014). The impact of Bolsa Familia on women’s decision-making power. World Dev.

[39] Meireles F, Andrade LVR (2017). Magnitude eleitoral e representado de mulheres nos municipios brasileiros. Revista de Sociologia Política.

[40] Macinko J, Dourado I, Aquino R, de Bonolo PF, Lima-Costa MF, Medina MG (2010). Major expansion of primary care in Brazil linked to decline in unnecessary hospitalization. Health Aff (Millwood).

[41] Presidencia da República de Brasil (2009). Lei N° 12.034, de 29 de setembro de 2009.

[42] dos Santos BC (2017). 5 dados sobre a participado das mulheres na política brasileira. Politize! [serial on the Internet].

[43] Bolognesi B, Monseff Perissinotto R, Codato A (2016). Reclutamiento político en Brasil: mujeres, negros y partidos en las elecciones federales de 2014. Rev Mex Cieñe Polit Soc.

